# Quintuple Arterial Minimally Invasive Coronary Artery Bypass Grafting: Non-touch Aortic Technique

**DOI:** 10.7759/cureus.93660

**Published:** 2025-10-01

**Authors:** Danko Grujic, Tatjana Kokotovic, Oliver Radmili, Vladimir Jakovljevic, Vojkan Aleksic

**Affiliations:** 1 Department of Cardiac Surgery, University Clinical Center of Serbia, Belgrade, SRB; 2 Department of Anesthesiology and Intensive Care, University Clinical Center of Serbia, Belgrade, SRB; 3 Clinic for Vascular and Endovascular Surgery, University Clinical Center of Serbia, Belgrade, SRB; 4 Department of Medical Physiology, University Clinical Center of Kragujevac, Kragujevac, SRB

**Keywords:** artery disease, bypass, coronary surgery, heart, minimally invasive

## Abstract

Minimally invasive coronary surgery has numerous benefits over conventional coronary surgery through sternotomy. Patients have less surgical trauma to tissue, a shorter stay in the hospital, and a better quality of life after the operation. In the presented surgical technique, minimally invasive cardiac surgery (MICS)-coronary artery bypass graft surgery (CABG), we achieved total arterial revascularization of the myocardium with five bypasses, off-pump, without manipulation of the aorta. Using left thoracotomy, the left internal mammary artery was harvested, and from the non-dominant hand, the radial artery (AR) was harvested with the no-touch technique. A composite T-graft was created with the left intrathoracic mammary artery (LIMA) and the AR. Then, the LIMA was used as a sequential graft to a diagonal (Dg) branch, and the distal LIMA was used for a termino-lateral (T-L) anastomosis between the LIMA and the left anterior descending artery (LAD). The AR was used for grafting obtuse marginal (OM) branches (first OM (OM1), second OM (OM2)) and the posterior descending artery (PDA), as a LIMA-AR-OM1-OM2-PDA graft, jumping anastomosis. The patient was discharged from the hospital on the fourth postoperative day, and after three months, a control computed tomography (CT) coronary angiography confirmed optimal graft patency. This surgical technique highlights the feasibility and benefits of total arterial, off-pump, non-touch aorta surgical revascularization, reducing the perioperative risk and promoting better functional recovery.

## Introduction

Coronary artery disease is the leading cause of death in developed, middle-income countries, and in recent years, it has increased in low-income countries [1]. The choice of treatment, especially for multivessel disease, is coronary bypass surgery [2]. Surgical revascularization of the myocardium has a long-term better outcome over percutaneous coronary interventions (PCIs), with the greatest benefit in multivessel disease [3]. Conventional surgery includes a medial sternotomy and cardiopulmonary bypass (CPB), and the procedure is performed in arrest. To minimize the invasivity of traditional coronary artery bypass graft surgery (CABG), reduce hospital days and the risk of infection, and maximize patient outcomes, minimally invasive cardiac surgery (MICS)-CABG was developed [4]. This type of surgical revascularization involves a small anterior left thoracotomy incision of about 5 cm, performed on-pump (involving a CPB machine) or off-pump (without a CPB machine). Off-pump surgery diminishes the complications of CPB, such as an inflammatory response, stroke, atrial fibrillation, and acute kidney failure [5]. Additionally, the non-touch aortic technique (without cannulation, cross-clamping, or proximal anastomosis) reduces the risk of short-term mortality, stroke, and bleeding [6]. Despite considerable benefits over the conventional CABG procedure, there is a steep learning curve, and the complexity of performing this operation prevents it from becoming routine practice [7]. In studies, typically, MICS-CABG is performed the most, with two, three, or four bypasses, and, rarely, with five bypasses [8].

In an assessment of the available literature, we did not identify studies reporting procedures that include all arterial grafts, performed off-pump, without touching the aorta, or with five bypasses (six anastomoses). In this case report, we present the off-pump MICS-CABG procedure, conducted with arterial grafts, and five bypasses for full revascularization of the myocardium, without aortic manipulation.

## Case presentation

Our patient was a 61-year-old male who had a non-ST-segment elevation myocardial infarction (NSTEMI). After coronary angiography and other diagnostics were performed, left anterior descending artery (LAD) with 80% proximal stenosis, medial with 80-90%, diagonal (Dg) branch with 95% stenosis, obtuse marginal (OM1) proximal with occlusion, OM2 with sub-occlusion stenosis, and right coronary artery (RCA) with medial occlusion and blood supply from the heterocolaterals were identified. The patient had the common comorbidities: diabetes mellitus type II, hypertension, and dyslipidemia. The laboratory analysis was satisfactory, and all vital parameters were within the reference range (Table 1).

**Table 1:** Vital signs and laboratory and echocardiography findings of the patient

Parameters	Results	References values	Unit
Blood pressure (systolic/diastolic)	130/80	80-120/60-80	mmHg
Heart rate	72	60-80	Beats/min
Respiratory rate	14	12-20	Breaths/min
Oxygen saturation	98	≥95	%
Body temperature	36.4	36-37	°C
Laboratory findings			
Hemoglobin (Hb)	139	138-175	g/l
White blood cell (WBC)	9.4	3.4-9.7	X10^9^/l
Platelet count (PLT)	179	158-424	X10^9^/l
Glucose level in blood	5.9	3.9-6.1	mmol/l
Serum creatinine	64	59-104	µmol/L
Total cholesterol	5.4	≤5.2	mmol/l
Low-density lipoprotein (LDL) cholesterol	3.1	≤3.0	mmol/l
High-density lipoprotein (HDL) cholesterol	˃1.0	0.86	mmol/l
Triglycerides	1.9	˂1.7	mmol/l
Echocardiographic parameters			
Left ventricular ejection fraction (LVEF)	52	55-70	%
Left ventricle end-diastolic diameter (EDD)	56	42-58	mm
Left ventricle end-systolic diameter (ESD)	30	25-40	mm
Left atrial diameter (LA)	50	30-40	mm

Additionally, echocardiography showed that the left ventricular ejection fraction (LVEF) was estimated at 52%, the left ventricular end-diastolic diameter was 56 mm, the end-systolic diameter was 30 mm, the left atrium was 50 mm, and the septum was hypokinetic (Table 1). An elective cardiac surgery procedure was planned. After preoperative anesthesiology medication, the patient was positioned on the right side and then placed in a double-lumen endotracheal tube. The incision was performed at the fourth intercostal space, and after opening the pleural cavity, a retractor was placed to harvest the internal mammary artery.

From the first to sixth ribs, the left intrathoracic mammary artery (LIMA) was harvested in a skeletonized fashion with a harmonic scalpel, under direct vision. After administering heparin, the distal end of LIMA was cut and coated with papaverine. At the same time, from the non-dominant arm, a radial artery (AR) was harvested using a harmonic scalpel, with a non-touch technique [9]. First, an anastomosis was made between the LIMA and AR, as an end-to-side "T" graft. Then, the LIMA was used as a sequential graft to Dg, and the distal LIMA was used for a termino-lateral (T-L) anastomosis between the LIMA and LAD.

**Figure 1:**
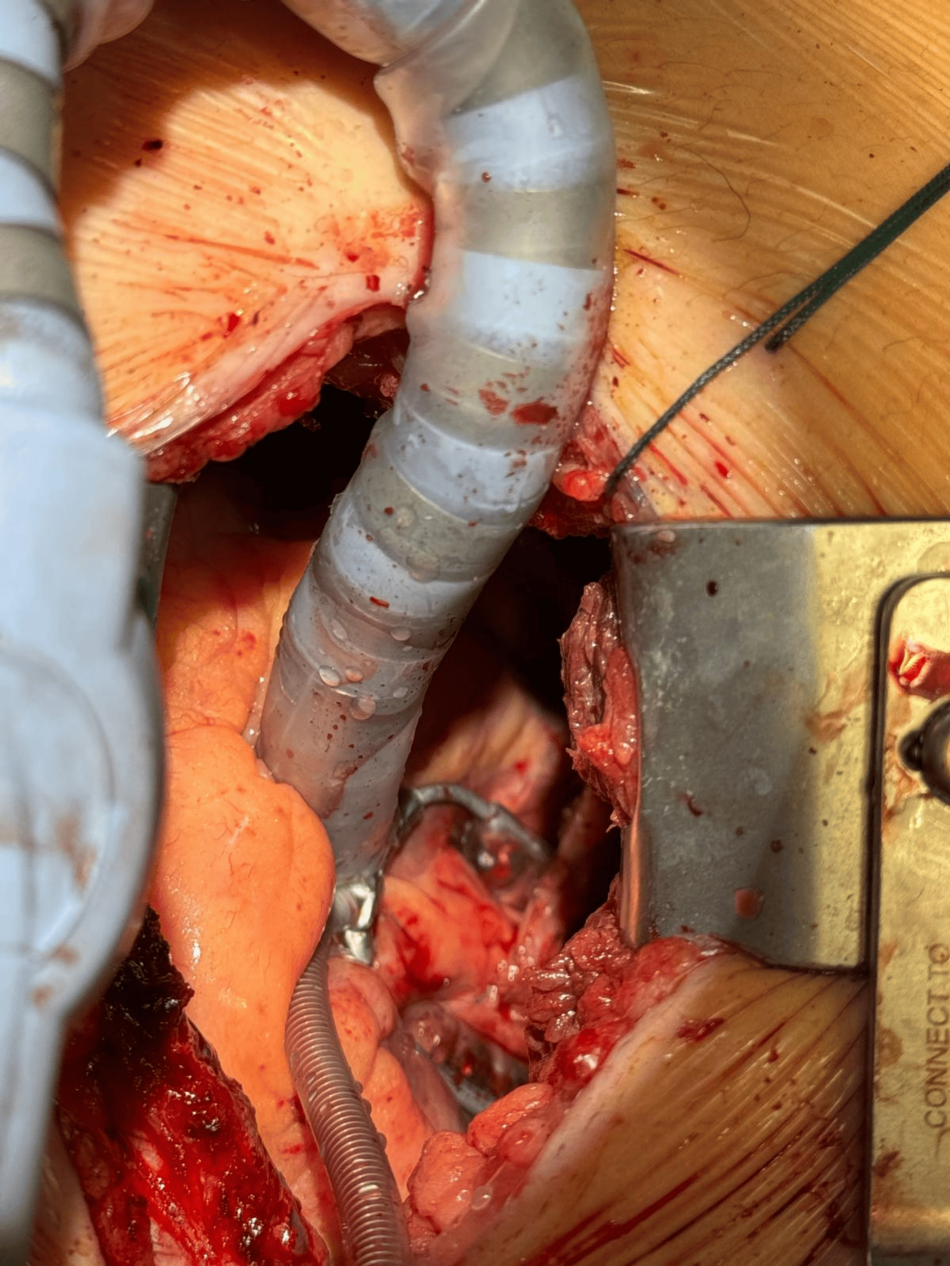
Anastomosis of the AR-PDA The figure shows an anastomosis between the radial artery (AR) and the posterior descending artery (PDA) performed as part of a sequential arterial revascularization plan.

By placing the suture through the pericardium and pulling it to the side of the chest wall, the heart was brought into position for grafting the obtuse marginal (OM) branch. The circumflex artery, the first OM (OM1) branch, was identified, and the suction stabilizer Octopus (Medtronic, Minneapolis, Minnesota, US) was placed to make the LIMA-AR-OM1 side-to-side anastomosis. Next, an anastomosis was made between OM2 and AR in the formation LIMA-AR-OM1-OM2. The distal part of the AR was used for anastomosis to the posterior descending artery (PDA) branch to make a LIMA-AR-OM1-OM2-PDA graft, jumping the anastomosis (Figure 1).

This procedure was performed without touching the aorta, and all anastomoses were performed off-pump (Video 1).

**Video 1:** A video showing MICS-CABG performed through a minimally invasive approach, thoracotomy The video demonstrated a step-by-step operative technique, Minimally invasive cardiac surgery (MICS)-coronary artery bypass graft surgery (CABG) through left mini thoracotomy. The procedures include the setup, incision, and harvesting of the left mammary artery. Then, a "T" graft was constructed between the radial artery (AR) and left intrathoracic mammary artery (LIMA), followed by construction jumping and sequential anastomosis. The whole procedure was off-pump, without touching the aorta.

**Figure 2:**
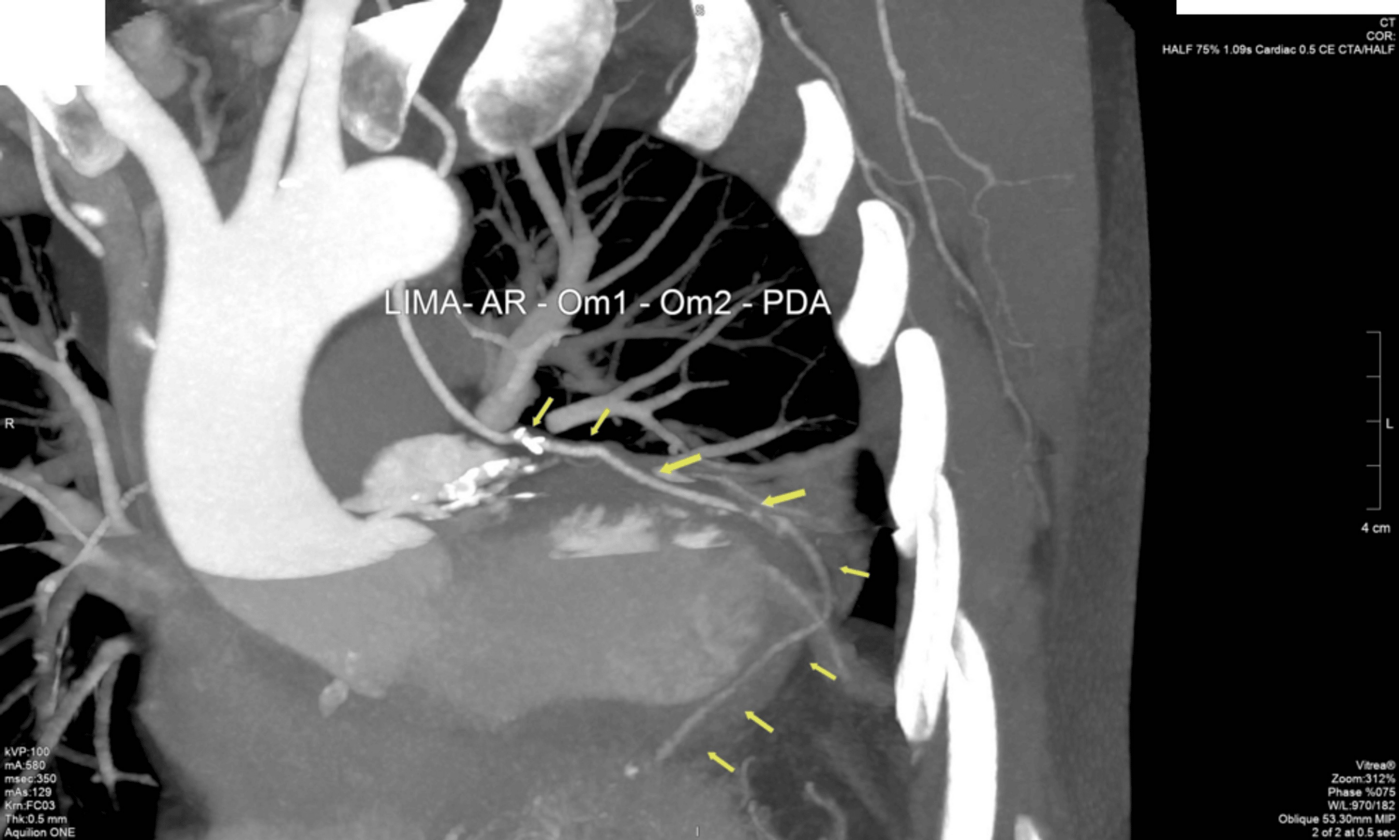
Three months after the operation, a control CT coronarography was performed. The postoperative CT coronary angography showed patency of the left intrathoracic mammary artery (LIMA) and radial artery (AR) used in the sequential anastomosis. The diagnostic tool verified adequate graft flow and successful revascularization.

**Figure 3:**
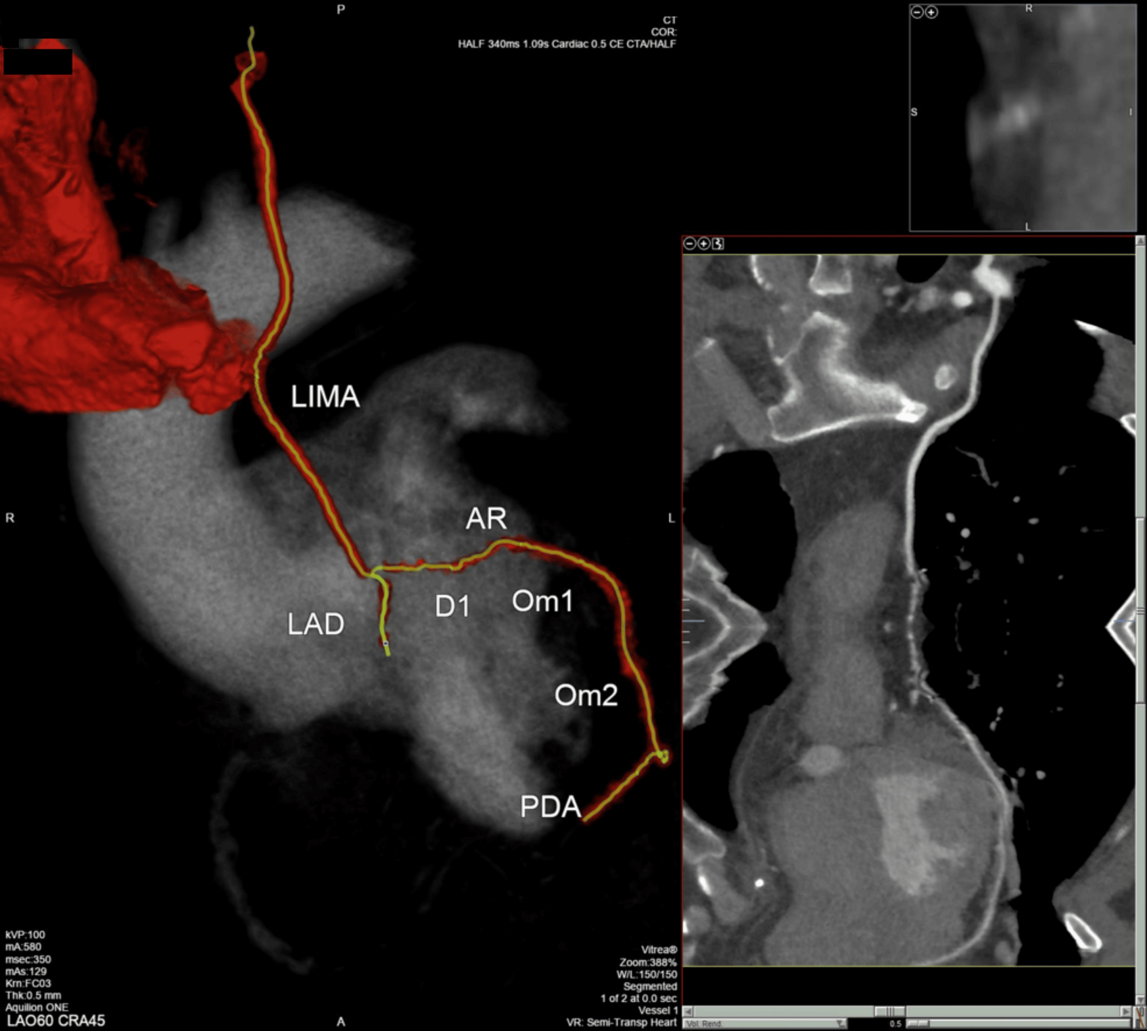
Three months after the operation, a control CT coronarography was performed. In this figure, sequential grafts (LIMA-AR-OM1-OM2-PDA) are visualized. Also, the anastomosis LIMA-DG-LAD is better reflected. LIMA: left intrathoracic mammary artery; AR: radial artery; OM1: first obtuse marginal; OM2: second obtuse marginal; PDA: posterior descending artery; DG: diagonal; LAD: left anterior descending artery

The drain was placed in the hemithorax, and the surgical wound was closed in layers. After the operation, the patient was stable from the cardiopulmonary viewpoint, and on the fourth postoperative day, was discharged from the hospital. Three months after the operation, on the control computed tomography (CT) colonography, all grafts had defined adequate flow (Figures 2, 3).

## Discussion

This surgical technique included complete revascularization with five bypasses in a patient with multivessel coronary artery disease. Total surgical revascularization of the myocardium was achieved with arterial grafts, illustrated in numerous studies with advantageous long-term postoperative outcomes compared to conventional venous grafts [10].

The main goal of total arterial revascularization is to achieve good long-term myocardial perfusion. This is possible only with arterial grafts. Moreover, technically, it is more demanding to perform this procedure than conventional CABG, and the surgeon must have extensive experience in minimally invasive surgery and off-pump surgery. A structural training program and consistent team members are required for this procedure. In the frame of minimally invasive surgery, the non-touch aorta and off-pump approaches effectively reduce the risk of complications. Wound-related infections and neurological complications are significantly lower in this approach [11]. The number of comorbidities or older age may increase mortality and morbidity, which can be reduced by minimally invasive total revascularization of the myocardium [12]. Moreover, minimally invasive surgery improves the patients' quality of life, accelerates recovery, reduces surgical trauma, shortens hospital stays, and emphasizes economic benefits [13]. This enables the patient to return faster to daily activities and reduces overall hospital healthcare costs. Additionally, patients' preference for this type of surgery points out the need for it to become a standard practice and increase the volume of such procedures in cardiac surgery centers. Still, there is a need for large prospective and randomized studies to compare minimally invasive and conventional surgical revascularization of the myocardium.

## Conclusions

This study highlights the possibility of a surgical approach, with the aorta not touched, and using only arterial grafts sutured off-pump for the complete treatment of multivessel disease. The beginnings of minimally invasive cardiac surgical revascularization mostly describe single-vessel disease, while the conventional approach is reserved for multivessel disease. The minimally invasive surgical technique with complete arterial revascularization of the myocardium represents a feasible option for patients with multivessel coronary disease. The combination of off-pump surgery and not manipulating the aorta emphasizes a safer procedure. Using arterial grafts has shown good long-term results. In addition, this procedure reduces mortality, morbidity, and healthcare costs. The short follow-up results were satisfactory in our study, but prospective long-term trials are needed for this to become standard practice in a surgical center.

## References

[1] Gaziano TA, Bitton A, Anand S, Abrahams-Gessel S, Murphy A (2010). Growing epidemic of coronary heart disease in low- and middle-income countries. Curr Probl Cardiol.

[2] Cavalcante R, Sotomi Y, Zeng Y (2017). Coronary bypass surgery versus stenting in multivessel disease involving the proximal left anterior descending coronary artery. Heart.

[3] Holm NR, Mäkikallio T, Lindsay MM (2020). Percutaneous coronary angioplasty versus coronary artery bypass grafting in the treatment of unprotected left main stenosis: updated 5-year outcomes from the randomised, non-inferiority NOBLE trial. Lancet.

[4] Sef D, Thet MS, Hashim SA, Kikuchi K (2024). Minimally invasive coronary artery bypass grafting for multivessel coronary artery disease: a systematic review. Innovations (Phila).

[5] Sá MP, Ferraz PE, Escobar RR (2012). Off-pump versus on-pump coronary artery bypass surgery: meta-analysis and meta-regression of 13,524 patients from randomized trials. Rev Bras Cir Cardiovasc.

[6] Zhao DF, Edelman JJ, Seco M (2017). Coronary artery bypass grafting with and without manipulation of the ascending aorta: a network meta-analysis. J Am Coll Cardiol.

[7] Fatehi Hassanabad A, Kang J, Maitland A, Adams C, Kent WD (2021). Review of contemporary techniques for minimally invasive coronary revascularization. Innovations (Phila).

[8] Liu JJ, Kong QY, You B (2021). Surgical challenges in multi-vessel minimally invasive coronary artery bypass grafting. J Interv Cardiol.

[9] Cabrucci F, Bacchi B, Chiarello B, Dokollari A, Bonacchi M (2023). Radial artery harvesting with harmonic scalpel: fully no-touch technique. Multimed Man Cardiothorac Surg.

[10] Dominici C, Chello M, Saeed S (2022). Outcomes of total arterial revascularization vs conventional revascularization in patients undergoing coronary artery bypass graft surgery: a narrative review of major studies. Pak J Med Sci.

[11] Sellin C, Asch S, Belmenai A, Mourad F, Voss M, Dörge H (2023). Early results of total coronary revascularization via left anterior thoracotomy. Thorac Cardiovasc Surg.

[12] Guo MH, Toubar O, Issa H (2024). Long-term survival, cardiovascular, and functional outcomes after minimally invasive coronary artery bypass grafting in 566 patients. J Thorac Cardiovasc Surg.

[13] Gong Y, Wang X, Li N (2022). A partially randomized patient preference trial to assess the quality of life and patency rate after minimally invasive cardiac surgery-coronary artery bypass grafting: design and rationale of the MICS-CABG PRPP trial. Front Cardiovasc Med.

